# Social response to the delivery of HIV self-testing in households: experiences from four Zambian HPTN 071 (PopART) urban communities

**DOI:** 10.1186/s12981-020-00287-y

**Published:** 2020-06-11

**Authors:** Chiti Bwalya, Musonda Simwinga, Bernadette Hensen, Lwiindi Gwanu, Able Hang’andu, Chama Mulubwa, Mwelwa Phiri, Richard Hayes, Sarah Fidler, Alwyn Mwinga, Helen Ayles, Virginia Bond, the HPTN 071 (PopART) study team

**Affiliations:** 1 Zambart, School of Public Health, University of Zambia, Lusaka, Zambia; 2 Department of Infectious Disease Epidemiology, Epidemiology and Population Health, London School of Hygiene and Tropical Medicine, London, UK; 3 Imperial College, London, UK; 4 Clinical Research Department, Infectious and Tropical Diseases, London School of Hygiene and Tropical Medicine, London, UK; 5 Department of Global Health and Development, Faculty of Public Health and Policy, London School of Hygiene and Tropical Medicine, London, UK

**Keywords:** HIV self-testing, Community health workers, Social harms, Door -to-door distribution of HIVST, Zambia

## Abstract

**Background:**

Door-to-door distribution of HIV self-testing kits (HIVST) has the potential to increase uptake of HIV testing services (HTS). However, very few studies have explored the social response to and implications of door-to-door including secondary distribution of HIVST on household relations and the ability of individuals to self-test with or without supervision within households.

**Methods:**

A CRT of HIVST distribution was nested within the HPTN 071 (PopART) trial, in four Zambian communities randomised to receive the PopART intervention. The nested HIVST trial aimed to increase knowledge of HIV status at population level. Between February 1 and April 30, 2017, 66 zones (clusters) within these four communities were randomly allocated to either the PopART standard of care door-to-door HTS (33 clusters) or PopART standard of care door-to-door HTS plus oral HIVST (33 clusters). In clusters randomised to HIVST, trained Community HIV care provider (CHiPs) visited households and offered individuals aged ≥ 16 and eligible for an offer of HTS the choice of HIV testing using HIVST or routine door-to-door HTS (finger-prick RDT). To document participants’ experiences with HIVST, Interviews (n = 40), observations (n = 22) and group discussions (n = 91) with household members and CHiPs were conducted. Data were coded using Atlas.ti 7 and analysed thematically.

**Results:**

The usage and storage of HIVST kits was facilitated by familiarity with and trust in CHiPs, the novelty of HIVST, and demonstrations and supervision provided by CHiPs. Door-to-door distribution of HIVST kits was appreciated for being novel, convenient, private, empowering, autonomous and easy-to-use. Literacy and age influenced accurate usage of HIVST kits. The novelty of using oral fluids to test for HIV raised questions, some anxiety and doubts about the accuracy of HIVST. Although HIVST protected participants from experiencing clinic-based stigma, it did not address self-stigma. Within households, HIVST usually strengthened relationships but, amongst couples, there were a few reports of social harms.

**Conclusion:**

Door-to-door distribution of HIVST as a choice for how to HIV test is appreciated at community level and provides an important testing option in the sub-Saharan context. However, it should be accompanied by counselling to manage social harms and by supporting those testing HIV-positive to link to care.

## Background

Knowledge of HIV status is an essential prerequisite to timely access to HIV treatment and prevention services. Achieving the first-90 of the UNAIDS ‘90-90-90’ target, which calls for 90% of all people living with HIV (PLWH) knowing their HIV-positive status [1], requires the use of new technologies and innovative strategies to deliver HIV testing services (HTS) to reach individuals previously not tested and encourage re-testing amongst those testing HIV-negative. HIV self-testing (HIVST) has the potential to move HIV testing from health facilities into communities and households. This can allow people to test themselves at times and places convenient to them thereby avoiding challenges faced at the health facility, such as long waiting time and stigma related barriers to HTS [2]. For this reason, HIVST is increasingly being perceived as a testing approach that can help the wider uptake of HTS and support countries to achieve the UNAIDS 90-90-90 targets [1, 3].

Early studies of HIVST in sub-Saharan Africa (SSA) have reported that it is highly acceptable as it overcomes significant barriers to clinic based HTS, such as privacy and confidentiality [4–8]. However, few studies have explored the social challenges and the implications of door-to-door distribution of HIVST on household relations and the ability of individuals to self-test with or without supervision. There is also a need for further research on the potential benefits of door-to-door distribution of HIVST, including secondary distribution, on individuals and their households. In addition, self and social harms, the implications of lack of counselling on individuals, and poor linkage following a positive test result are potential concerns that require further investigation [9].

Zambia has made good progress towards improving access to HTS by increasing the number of facilities providing HTS. By the end of 2016, there were over 1, 800 health facilities countrywide that were providing HTS [10].

The implementation of several initiatives, coupled with the availability of ART, have also helped to increase the number of people testing for HIV. These initiatives have included the introduction and use of rapid tests, opt-out provider initiated testing, integration of HIV testing with other services such as sexually transmitted infection (STI), tuberculosis (TB), family planning and antenatal clinics, use of community based HIV testing methods (including ‘door-to-door’ approach), as well as weekend HIV testing appointments for men [11–14].

Despite this increase in the coverage of HTS services, an estimated 33.9% of PLWH in Zambia did not know their HIV status by 2017 [15]. Uptake of HTS has been reported to be lower in men and adolescents [16, 17]. Barriers to accessing HTS include perceived health workers’ inability to maintain confidentiality, fear of HIV-related stigma, direct and indirect financial costs related to accessing HTS, inferring HIV status from a partner’s status, misconception of HIV testing, the fear of a positive result and gender inequality [12, 18]. Mobility driven by livelihood options and alcohol use also undermine uptake of home-based HTS [14, 19]. Many HIVST studies have been quantitative in design, with supporting qualitative enquiry focusing on feasibility, usability, preferences and acceptability of HIVST [2, 9]. Insights into how individuals actually use HIVST within a home setting, the social implications of this use and secondary distribution to other household members are unusual. Further, there is limited literature on preferred mode of distribution, storage of door-to-door delivered test kits, the role of lay counsellors in distributing and the social risks of HIVST. Focusing on how HIVST was experienced through a particular household distribution approach in four high-density urban Zambian communities, this paper highlights the household and community response to door-to-door distribution of HIVST kits.

## Methods

### HIVST CRT Design and context

This qualitative study was a component of a cluster randomised trial (CRT) of community-based distribution of HIVST [20], nested within HPTN 071 (PopART), a larger CRT that evaluated the impact of a door-to-door universal testing-and-treatment (UTT) intervention on HIV incidence at population-level [20–22]. The HISVT CRT was implemented in the third year of the HPTN 071 (PopART) intervention delivery. Details of the nested CRT and the main HPTN 071 (PopART) trial intervention have been described elsewhere [20–22].

Briefly, this nested HIVST CRT was implemented for 3 months in four Zambian communities randomised to receive the PopART intervention [20]. The four communities were divided into 66 zones (geographical areas referred to as ‘clusters’ in the HIVST trial), which were randomly allocated (1:1) to either the PopART standard of care door-to-door HTS alone (33 clusters) or PopART standard of care door-to-door HTS plus oral HIVST (HIVST group; 33 clusters) [20]. In clusters randomised to the HIVST group, existing experienced lay counsellors, called Community HIV care Providers (CHiPs), were trained for additional 3 days on how to offer HIVST as an additional option for HIV testing and how to demonstrate use of an oral HIVST kit.

During household visits, CHiPs in the HIVST group offered individuals aged 16 years or older and consenting to participate in PopART and eligible for HIV testing a choice of HIV testing using oral HIVST or a finger-prick sampling of whole blood and rapid HIV testing (finger-prick RDT), which was done according to PopART procedures and the Zambian national HIV testing algorithm [20, 21]. Post-test counselling suitable for clients testing HIV positive or negative was provided by the CHiPs if results were shared with them.

The CHiPs also facilitated active linkage to care at the nearest government health facility for all clients who tested HIV-positive through follow-up home visits for clients who did not link to care and by escorting individuals who wished to be accompanied. Individuals choosing HIVST, could choose supervised HIVST in the presence of the CHiP or unsupervised HIVST in the absence of the CHiP. Regardless of how people chose to self-test, CHiPs demonstrated how to use the kit and how to read the result with the level of supervision and support provided being dependent on individual preference. HIVST was conducted in private spaces decided by the client either inside the house or in spaces outside the house that provided some privacy. In addition, clients who wished to test on their own were at liberty not to share their results with Chips.

Individuals aged 18-years or older who had a spouse/partner, who were resident in the same household but were not present during the CHiPs visit, were offered an HIVST kit for secondary distribution to the absent partner. For individuals choosing unsupervised HIVST or taking an HIVST kit for secondary distribution, CHiPs conducted follow up visits within 7 days of leaving the test kit at the household to verify use and offer follow-up services. CHiPs also provided a card with their phone number to allow the absent individual to contact them should additional support be required and for linkage to confirmatory testing and care services. Individuals opting for HIVST could either give the used HIVST kit back to the CHiP or return it confidentially at the local clinic, where the study team placed a box to facilitate return of used test kits.

### Study setting

This study was conducted in four high-density urban Zambian communities. For purposes of anonymity, the four communities are given codes from 1 to 4. Communities 3 and 4 were close to two district town centres and communities 1 and 2 were on the outskirts of a district town. Infrastructure common to all communities included a government health facility, primary schools, police stations, churches and recreational facilities (such as football pitches and drinking places), market areas and transport depots. Housing in all four communities was a mix of informal, poorer quality housing and formal, better quality, planned housing.

The distribution of housing type and socio-economic class varied across the communities. Women and young girls were more often found in households while young men were often seen out and about at transport depots, bars and other recreational facilities. Alcohol consumption was pronounced with more men drinking than women on a daily basis. Formal employment options were very limited, and the main livelihood option for most residents was trading in goods, with some household members leaving early in the morning and only returning late in the evenings. Many women worked in local markets. HIV testing and treatment services in all four communities were mainly provided by government facilities that many community members say are heavily congested [23].

### Recruitment of participants and data collection

Qualitative data were collected between February and May 2017. The data collection activities were carried out by three social scientists (CB, LG and AH) with technical support from senior social scientists (MS and VB).

In each community, at the beginning of the intervention, observation of the door-to-door distribution of HIVST, follow ups on test-kits left for absentee partners and support visits by CHiPs were carried out using a structured observation guide.

Additional observations of social and physical differences between zones, mobilization activities and drop off points for used test kits located at health facilities were also conducted. The number (n = 22) and type of observations are summarised in Table 1. Towards the end of the HIVST intervention, in-depth interviews (IDIs) with 40 purposively selected individuals who had either chosen or not chosen to self-test were conducted, including with mobile and hard-to-reach individuals that are rarely found at home due to daily mobility linked to livelihood options.

**Table 1:** Methods, number and characteristics of participants

Data collection activity	Category of participants	Gender		Total
		Male	Female	
FGDs	CHWs (CHiPs)	12	20	32
	Men	19	–	19
	Health committee members	11	29	40
	Total # of participants for FGDs	42	49	91
IDIs	*Participants choosing HIVST*			
	Adults	5	7	12
	Adolescents (16–19 years old)	3	5	8
	Couples	2	2	4
	Hard to reach populations			
	Traders and other busy individuals	3	1	4
	Formally employed	1	1	2
	Sex workers	0	1	1
	MSM	1	0	1
	Substance (alcohol) user	1	0	1
	Total # of participants choosing HIVST interviewed	16	17	33
	*Participants not choosing HIVST*			
	Adults	0	4	4
	Adolescents	1	2	3
	Total # of participants not choosing HIVST interviewed	1	6	7
	Grand total of all participants interviewed	17	23	40
Observations	Community spiral walks			4
	CHiPs delivery of HIVST in homes			11
	Secondary distribution follow up observations			8
	Total # of observations conducted			22

Focus group discussions (FGDs) with two separate purposively selected groups of men and CHiPs were also conducted in each community. A total of 19 men and 32 CHiPs participated in the FGDs (see Table 1).

IDIs and FGDs were conducted in either Bemba or English and at the health facility or convenient and private locations chosen by participants. The IDI and FGD guides focused on experiences of households with HIVST and social implication of a door-to-door distribution approach of self-test kits on household social relation including that of couples. In addition, the guides also explored other potential modes of distribution of HIVST, role of CHiPs in distributing HIVST, response of different groups to HIVST (men, women, young people, and substance abusers) and social harms linked to HIVST.

### Data management and analysis

Field notes taken during observations, IDIs and FGDs were written and typed in Microsoft Word immediately after each data collection activity. Later, verbatim quotes were retrieved by listening to the IDI and FGD recordings. The observation and summary reports were reviewed by the first author for accuracy and quality. Using a thematic analysis approach, a codebook with corresponding code definitions using deductive and inductive approaches was developed [24]. All written up notes from observations, IDIs and FGDs were coded and managed using Atlas.ti version 7. The coded data outputs from Atlas.ti 7 for specific themes were then produced and shared amongst the social scientists at an analysis workshop where they were read and discussed followed by writing up themed summaries that acted as units of analysis.

### Ethics approval and consent to participate and for publication

Ethical approval for the study was granted by the London School of Hygiene and Tropical Medicine (LSHTM) Ethics Committee and the University of Zambia Biomedical Ethics Committee (UNZA BREC). Permission to do the study was also granted by the National Health Research Authority and the Zambia Medicines Regulatory Authority.

Written informed consent was obtained from all participants interviewed and those taking part in FGDs. In case of non-literate participants, a witnessed thumb-printed informed consent was obtained. All participants gave written consent to participate in the study including giving consent to the following statement: ‘I agree that anonymised direct quotes from the interview, group discussion or observations field notes may be used in the public reporting of findings from this study’. Additional verbal consent and permission was sought from all individual participants in households before the observations were made. All participants were interviewed from private locations of their choice. To ensure confidentiality, all personal identifying information such as home address and phone numbers were removed from the data transcripts. In addition, names of study communities and participants were also anonymised.

## Results

We first describe participants’ views about their preferred HIVST distribution places. We then describe the observations and experiences of households with the door-to-door HIVST distribution, including the role of CHiPs. The impact of HIVST on social relations within the households is described in the last component of the results.

### Participants’ suggested distribution places and networks

Although the trial focused on door-to door-distribution of HIVST by CHiPs, participants were asked to suggest places and social networks through which HIVST could be delivered in their community. Their suggestions mirrored gendered spaces (i.e. both women and men chose spaces where they are mostly found), livelihood activities, and social networks of people they mostly interact with (Table 2). For example, women suggested antenatal clinics, water points and local markets.

**Table 2:** Participants’ suggested places for distribution of HIVST kits

Participant type	Distribution places and networks
Men and other hard-to-reach groups	Workplace, fishing camps, bars, sports stadiums and other recreational places. Homes of CHiPs, family, friend and spouse networks
Women	Home, antenatal and under-five clinics, water points, fishing camps, the market place, church, family and friends’ networks
Adolescents	Youth clubs, school anti-AIDS clubs, education institutions, mobile outreach initiatives, football grounds and other sports and recreation facilities. Home for young women with approval from parents

Most participants desired for the distribution-points not to have procedures and process similar to those found at health facilities that bring about long waiting time and stigma. To facilitate counselling and support, participants suggested that CHiPs should manage community HIVST distribution points.

### Door-to-door distribution of HIVST

Observations around door-to-door distribution of HIVST showed that the rapport created between CHiPs and household members in the preceding 2 years of the PopART intervention facilitated the acceptance of HIVST in the households. The field notes below portray this observation:

‘*CHiPs are very well known and established in this zone. I noticed they greeted almost everybody along the way. The households we visited welcomed us very well with bright smiles and warm greetings. They also gave us seats and showed a lot of interest. For instance, the wife at the first household was seated chatting at a neighbour’s house, (but) immediately she saw us in her yard, she excused herself and came to meet us with delight in her countenance and offered us seats’ (Door*-*to*-*door distribution of HIVST observations field notes, community 2, 20170216).*

HIVST was perceived as a novel and interesting testing method. Not surprising, these communities had a long history of HIV testing using the finger-prick RDT method. A 36-year-old woman, who had previously tested using the finger-prick RDT method, said she chose HIVST because she wanted to *“try the new thing*”.

HIVST was appreciated by a sex worker, heavy drinker, teacher, mineworker and trader who all said that *‘it was better to test oneself’*. Individuals belonging to these sub groups and many men said that they have little time to go to the clinic because of their work and linked mobility. Indeed, women accepting HIVST kits for secondary distribution to their partners often mentioned that their husbands were not opposed to testing but just too busy to go to the health facility. Men from two FGDs reflected on how door-to-door delivered HIVST enabled them to test at an appropriate time and did not disturb their work schedules:

*“The tests are convenient because people can test in their own time. It is difficult [for counsellors] to talk to traders, drivers and other people when they are working. They [traders, drivers* etc*.] can’t really concentrate because of work…. A trader will be busy organizing their market stand and keeping watch for potential customers when a counsellor is trying to deliver the message on testing. But with self*-*test kits, the working class, traders, drivers, farmers can access the kits through their partners and test in their own time” (FGD, men, Community 1).*

Following the CHiPs explanation and demonstration of kit use, most participants interviewed about or observed using the kit understood the oral and written instructions accompanying the kit, and read and interpreted their test results:

*“Self*-*testing … is as simple as (counting) 1,2,3, there was nothing difficult about it even if you gave me the test again, I can still test myself” (male trader, supervised tester, community 3).*

HIVST was considered to be painless, and this was highlighted as one of its advantages over finger-prick RDT by most individuals.

In one observation, an 18-year-old man who chose to self-test said that he ‘*dreaded the pain*-*inflicting lancet*’. A degree of empowerment also arose from knowing how to conduct a self-test and reading one’s result. In addition, HIVST was seen as a strategy where no “*swapping*” of results could occur, a problem perceived to sometimes occur at the health facility for people being tested by facility staff.

There were mixed feelings about the accuracy of HIVST. Although most individuals with an HIV-positive result trusted the results and accepted having a confirmatory blood test, some used the need for a confirmatory blood test to throw doubt on the accuracy of HIVST. Some participants questioned the accuracy of HIVST because HIV is found in blood and not in saliva and the mouth. One elderly man, when talking to CHiPs, asked whether people would be getting infected from kissing:

*“But people are becoming disturbed in a way that they are now thinking the virus is now found in the mouth and they are saying they will stop kissing…For you to give us test kits that utilise fluids from the mouth means the virus is present in the mouth” (FGD, CHiPs, Community 1).*

The convenience and control over where one can test from and time that the home environment and HIVST provided was widely appreciated. Many participants mentioned *“privacy”* and *“confidentiality”* as benefits of HIVST. Being able to test in one’s own bedroom and home was not only convenient, it also meant that no-one else saw the results unless the tester wished them to.

The hard-to-reach individuals such as traders, heavy drinkers, sex workers said that HIVST enhanced their sense of control; *“seeing things happen”* in private “*without anybody there”*. For couples who chose to test together, the confidentiality that HIVST provided was particularly valued. One couple who tested together using HIVST stated that testing together and sharing results was important so that they could know each other’s status and take better care of one another.

CHiPs were generally considered to uphold confidentiality, with CHiPs themselves recognising that confidentiality was important, especially as some CHiPs were themselves local residents. One CHiP reflected:

“*If you are known to spread information (rumours), people will refuse [to test for HIV at home]. But if a good report follows you, the people will not mind where you come from” (FGD, CHiPs, community 1).*

According to participants, privacy and confidentiality provided by HIVST delivered at home contrasted with the “*fear of being seen”* testing at health facilities, exacerbated by congestion and testing for the first time. This is reflected in the discussion with men:

*“What makes other people shy to go to the clinic for HIV testing is that people in the community talk too much about people who are HIV positive which makes them uncomfortable. As a result, many people do not go to access HIV testing at a place where there is a lot of people…testing alone [using HIVST] is better for certain people who fear being seen by community members that they accepted HIV testing” (adult men, FGD, Community 6).*

Participants referred to the risk of being talked about, being laughed at, being called names, and “*spreading (false) information”* against them if they were seen to test for HIV. However, some participants wondered how they would manage on their own if they tested HIV positive through HIVST. One adolescent felt that HIVST would facilitate denial around a positive test result and even encourage people with HIV to spread HIV to others through unprotected sex.

There were challenges observed with low literacy levels particularly to do with recording and interpretation of results. Some participants were concerned about the ability of individuals with low literacy to use the kit. Although such individuals often recalled instructions given to them and thus correctly performed the test, they could not always correctly read and record the results on a result slip that was included in an envelope as part of the HIVST kit. Individuals with higher literary recalled finding it easier to test without assistance from the CHiPs but the less literate and older participants were more likely to need support and supervision. Waiting for results was an anxious moment for some participants. Some participants fixedly watched the results emerge; one client shivered whilst waiting and another ululated. A few participants found it hard to wait for 20 min and read the results before the recommended time, while others waited for longer than 20 min. The two lines on the test result, one demonstrating the test had worked and the other giving the HIV result, confused some participants. For example, during one observation, one client interpreted an HIV-positive result as evidence that the test had worked and interpreted the result as HIV negative. This interpretation was corrected by the supervising CHiPs.

Most individuals who accepted HIVST kits for distribution to their spouse stored the kits in handbags or wardrobes and cupboards before and after use, sometimes moving them from one place to another after use as reflected in the following observation:

*‘A wife said she kept the unused kit in the cupboard but her husband moved it into the bedroom and hung it in a plastic bag against the wall after use’ (Door*-*to*-*door distribution of HIVST observations Field notes, community 2, 20170222).*

After HIVST, most individuals re-packed the used kits and made appointments with or waited for CHiPs to come and collect the kits while individuals not usually found at home often left the kits with their partners for return to the CHiPs.

*‘All the absentee clients’ wives during follow ups did not throw away the kits contents but repacked them in the test kit pouches, zip locked and eventually put it into the envelopes provided. These were later collected by the CHiPs who had promised they would return to collect them’ (Door*-*to*-*door distribution of HIVST observations field notes, community 2, 20170328).*

In a FGD, men said they preferred CHiPs to collect the used kits arguing that dropping them off into boxes provided for at the health facility was *“too exposing”* as individuals could easily be seen dropping the kits off.

### The impact of HIVST on household relationships

Introducing HIVST into a household had varied implications on household relationships. Discussions with CHiPs revealed that HIVST promoted happy and harmonious spousal relationships, especially for women that managed to get their husbands to test. Observations in a number of homes showed that women who accepted to get HIVST kits for their husbands believed that their husbands would accept to test when offered. During observations of secondary distribution follow up visits, wives said they were careful to choose a conducive time to introduce the HIVST kit to their husbands. A few wives had to ask their husband for permission before accepting HIVST from CHiPs. Follow up observations also showed that men often accepted testing when offered the kits by their spouses:

*‘In the two households where the husbands tested, the wives told us that they {their husbands} were very happy to have had the opportunity to test in their own time. They were thrilled about this option. In the first household, the couple tested after two days because the weekend was the only free time they had to test without rushing. In the third household, the husband tested at night, around 21:00* *h after he had eaten, rested from his day’s labours and was relaxed’ (*Secondary *distribution Follow ups observations Field notes, Community 2, 20170217).*

One man who was a miner and was absent from home most of the time said HIVST had a positive effect on his relationship with his wife. He narrated how she even bought him opaque beer, an alcoholic beverage produced through fermentation of a starch source or malt using water, sugar and yeast, in appreciation for testing. Other men said they tested out of obligation to lead by example as heads of households whiles others said they had not been pressured into making the decision in any way.

Some women explained that they did put pressure on their husbands to test for HIV. However, CHiPs expressed concern for men who drink excessively. The CHiPs said they had heard that some women intended to test their drunken husbands in their sleep:

*“Husbands, especially drunks, whose wives collect kits for them but refuse to test are at risk of being swabbed in their sleep. Some wives have even been heard saying it in the community” (CHiPs FGD, Community 1).*

Being pressurised or coerced to test by a spouse contrasted with being forced to test by others in the household. This was experienced by some women and young people, with men or older people exerting their authority in the household to insist that they test for HIV using an HIVST kit. A man forced his wife to test saying she had no choice as long as he was her husband. Some young people who were forced to test by their relatives said this did not disrupt their relationships. One young woman who was forced to test by her brother mentioned that there was nothing that had changed about their relationship even if she did not want to test initially. Another young man who initially did not want to test mentioned that there was no problem for him and his family after he tested.

The few documented cases of HIVST negatively impacting on household relations were amongst couples. Such cases included emotional distress, invasion of privacy, threatening and actual violence and separation. Emotional distress for those testing HIV-positive was a common experience for some men and women, especially in sero-discordant relationships were there was blame around who infected whom. Two cases of temporary marriage separation occurred when one partner blamed the other of infidelity and *“bringing HIV in the home”.* Follow-ups with these two cases revealed a history of alcohol abuse, gender based violence and lack of trust.

Counselling and education were frequently mentioned as a way for preventing social harms. The door-to-door distribution approach was also seen as an intrinsic mechanism for detecting social harms because of its potential to allow people to express themselves. It also allowed health workers to come closer to individuals.

According to the CHiPs, confirmatory testing and linkage to care for individuals testing HIV-positive through the supervised approach was much easier compared to individuals testing through the unsupervised approach. The latter were often clients who were difficult to find at home. In addition, CHiPs worried that such clients were also less likely to be open about their HIV status and link to care of their own volition. This is captured by the follow up visit observations field notes:

*‘This particular client [unsupervised tester] was not linked to care…. another follow*-*up visit was made. The client did not come to the clinic on the date agreed together with the CHiPs. After weeks, the client still did not come for treatment. CHiPs were making arrangements to do another follow up visit’. (Secondary distribution follows up visit observations field notes, community 4, 20170124).*

The majority of participants said counselling was important for providing support to people testing positive and to facilitate linkage into care. Counselling was also valued because it provided information clearing misconceptions around HIVST.

Respondents identified various counselling options appropriate for HIVST. These included face-to-face for individuals or couples, and telephone counselling. According to participants, unmarried individuals should be counselled alone and the married counselled as couples.

Being counselled by ‘strangers’ or professional counsellors was preferable to being counselled by known individuals. Family counselling was considered by many to be more *“tricky”* due to stigma, and “traditional” counselling (counselling by elders in the extended family) carried the risk of breaches of confidentiality. For example, one adult man said both family and traditional counselling *“could lead to rumour mongering and spreading private information”*. A few respondents were more in favour of family counselling saying it would facilitate care and support.

## Discussion

This study explored community opinions of and experiences with and the social implications of a door-to-door distribution approach of HIVST as an additional option for HIV testing in four high-density urban communities in Zambia. Door-to-door distribution of HIVST through CHiPs was appreciated by many participants for being novel, painless, bloodless, and easy to use. The privacy and confidentiality provided were widely appreciated along with the convenience of avoiding waiting times and exposure at the health facility and fitting testing around other daily demands and mobility. Literacy and age influenced ability to use the HIVST kits accurately and there was concern expressed by users about the reliability of the results. Door-to-door distribution of HIVST consolidated relationships in the household including couple relationships, although this was not always the case among sero-discordant couples. This mode of distribution resulted in a few cases of forced testing, disrupted relationships and social harms among couples, which were often embedded in a history of gendered and hierarchical social relations and strained relationships. Counselling was considered necessary to identify and deal with social harms as well as provide linkage to care for those testing HIV-positive.

CHiPs played a critical role in making HIVST acceptable. The rapport developed prior to the HIVST trial facilitated developing the trust necessary for the implementation of this community-based intervention. For years, households were only exposed to finger- prick RDT HTS making HIVST new, interesting and for some, a strange way of testing. CHiPs had a reputation for upholding confidentiality of participants’ status, something that participants said was lacking at the health facility as reported by in other studies [12].

Test kits were provided in private spaces making participants comfortable to test or accept the kit for later use. In other studies of community-based HIV testing services, privacy and confidentiality that came with this mode of distribution positively influenced acceptability [25–29].

In addition, this study found that acceptability was high because HIVST was a valued option that provided not only greater privacy, but ownership, control and convenience, findings consistent with prior research on HIVST [25, 26, 30, 31]. The broader generalizability of door-to-door distribution of HIVST led by lay counsellors needs to be explored further, although many communities do benefit from having lay counsellors. Currently many communities do not have a cadre of lay counsellors (like CHiPs) who visit every household systematically to discuss and offer HIV education, testing and linkage to care.

Supervised or unsupervised HIVST was said to be “easy” for many participants. However, detailed demonstrations by CHiPs masked the low literacy factor. The ability of individuals to self-test is partly dependent on literacy levels and previous exposure to HIV testing, but can be optimized through a demonstration of use and clear instructions [32, 33]. Similarly, in this study, for many, the use of Instructions For Use (IFU) with illustrative pictures, the use of flip charts and demonstration of use by CHiPs made the process of self-testing easier.

There were, however, a few cases where individuals were observed having difficulties with the testing process.

This suggests that for illiterate first time testers, supervised testing should be encouraged while re-testers with previous self-testing experience could conduct unsupervised testing. In South Africa, first time self-testers were found to experience similar challenges such as struggling with swabbing, opening the kit and interpreting of results when testing for the first time while re-testers had fewer challenges with the testing process [31].

In addition, evidence from cognitive interviews conducted as part of the Self-Testing Africa (STAR) study in Zambia showed that some people using the test kit for the first time made mistakes at critical stages of the testing process such as specimen collection [33].

Stigma (the fear of being seen testing at the health facility) and other factors, such as perceived lack of confidentiality associated with health facilities, impede uptake of facility based HTS [12, 34]. HIVST has been cited by others as a testing method that can reduce experiences of facility-based stigma [9]. In this study, anticipated stigma was the most common type of stigma that participants talked about. This was considered the main deterrent to testing at the health facility and made HIVST more attractive. However, a few participants also felt that HIVST would not allow people who tested HIV-positive to talk about and address HIV stigma more directly and could easily increase internalised stigma. Further, couples had a strong preference for CHiPs picking up the used test kits to avoid the potential gossip and inconvenience of dropping off used kits at the health facility. These findings suggest that there is risk that HIVST, while reducing one form of stigma (health facility based), has the potential to actually increase another form (self-stigma).

Therefore, active stigma reduction strategies are needed in communities to help PLWH overcome self-stigma and empower them to get to the health facility for confirmatory testing and linkage to care.

For many individuals, especially mobile men, the convenience that came with door-to-door delivered HIVST and secondary distribution was appreciated. HIVST made accessing HTS easier and quicker, leaving them with enough time to get back to their work or business, and then test later at their own convenience. In most sub-Saharan Africa countries, men are less likely than women to test for HIV, and are therefore less likely to know their HIV positive status and link to care [13]. Mobility linked to livelihood has been cited as a key barrier for testing [19, 35, 36]. Studies on facility based secondary distribution have shown that the approach is effective at reaching men [37, 38] Indeed, Mulubwa et al’s quantitative analysis of the HIVST trial nested within the main PopART study demonstrated an increase in men’s uptake of HTS in places where HIVST was made available [20].

HIVST also helped couples to test together resulting in improved relationships and creation of enabling environments for disclosure. These findings reaffirm what others have found, showing that secondary distribution is acceptable and can improve uptake of testing among couples [39]. This implies that gender and social relations within a home should be considered when offering test kits to women for secondary distribution to avoid social harm. Furthermore, women should be counselled by lay workers on how to cautiously introduce a test kit to their partners. Studies in Kenya and Uganda on secondary distribution through pregnant women showed high acceptability but emphasised the need for support to women and careful communication whenever a HIVST kit was being introduced to a male intended user [40, 41].

It should also be noted that, in this study, high acceptability of HIVST through secondary distribution may have been partly due to CHiPs cautiously counselling women on how to introduce test kits to their partners.

While most participants said that offer of HIVST improved their social relations with other family members, a few social harms (namely, invasion of privacy, deceit, forced testing, threatening violence or actual violence, emotional distress and separation of couples) were recorded. Finding from this study are similar to that of a review of evidence on social harms that found very few cases of social harms occurring in the practice of HIVST [41].

Other studies on HIVST have reported similar harms to those we identified [5, 42, 43]. However, our findings add to the literature on actual experienced social harms as most studies on harms have been on perceptions and not real life experiences from communities [9]. While these cases could ordinarily have also occurred under more traditional HTS, it is important to note that HIVST made women to have a greater influence and control over their partners’ testing process so much that they were able to persuade, induce, and even deceive them. HIV self-testing brought HIV testing into homes thereby fitting into ways of women coercing husbands, as well as challenging existing power relations.

Counselling was also seen as an intrinsic mechanism for detecting social harms. Many studies have highlighted the perceived challenge that may result from HIVST such as the need for post testing counselling, especially for those with positive results who need linkage to confirmatory tests and care [30]. Follow up visits by CHiPs provided an opportunity for post testing counselling, confirmatory testing and linkage. Future interventions using door-to-door distribution should embrace this strategy.

## Conclusion

Our study adds to evidence that HIVST delivered in people’s homes by lay counsellors is acceptable, including to harder to reach individuals, couples, men, and key populations. In addition, we show that secondary distribution of HIVST kits through partners can supplement door-to-door distribution by reaching mobile and busy men. This secondary distribution was enhanced by clear instruction, additional support from partners and counselling (remote and face to face) from CHiPs. However, our study also cautions that HIVST should be used with care to avoid social harms.

CHiPs were crucial for demonstrating how to use the kit, supporting safe and ‘correct’ usage and for providing counselling and linkage to care support to those newly diagnosed HIV-positive. How HIVST can be scaled up in the absence of the community health care providers is critical for long-term programmatic implementation to reach universal knowledge of HIV status and impact HIV incidence.

### Study limitations and strengths

The strength of this study is that it did not rely on perceptions but lived experiences of households and individuals with door-to-door distribution of HIVST. Triangulating findings through the use of interviews, FGDs and observations make the inquiry much stronger. For example, the use of observations provided researchers with first-hand information on how usable the HIVST kits were and factors affecting this. In addition, follow up observations provided information on household context and lived experiences of households after the door-to-door distribution and use of test kits. The use of interviews enabled us to include hard-to-reach participants both those refusing to test and accepting.

We acknowledge a number of potential limitations. First, views and experiences collected from a small, purposively selected sample of participants especially the hard to reach individuals may not necessarily represent views from all men, women and adolescents in all settings. However, our findings are transferable because we have provided a rich description of our study setting and can be used to explain experiences of households with door-to-door delivered HIVST in urban setting that are similar to our study sites. The other limitation is that, apart from the men that took part in the group discussion, we interviewed very few men and for those men accessing kits through secondary distribution, most of the views that were collected were reported speech from the wives or partners as we could not find most of the men at home to tell us their first-hand experiences with the test kits.

Talking to most of the men themselves or with their partners as couples would have identified more masculinity norms associated with accepting the kits from their partners which could have explained reasons for accepting or not accepting the kits. Despite this limitation, triangulation of data helped to fill in this gap as we talked to men in group discussions, conducted delivery and follow up observations, and we also talked to CHiPs who have had first-hand interaction with more men that either tested or did not test. The other limitation was that this study recorded very few social harms and this might reflect reporting bias if social-cultural beliefs of participants discourage the discussion of marital problems with outsiders. This may have made it difficult for couples to open up and share with CHiPs. Although the CHiPs had some experience with reporting incidents arising from the conduct of the main study, HIVST related social harms were a totally new area in terms of scope and focus.

### Implications

HIVST has the potential to reach individuals not being reached by conventional testing approaches and can help countries achieve the first of the UNAIDS 90-90-90 targets. As countries move toward integrating HIVST into national policies and regulations, additional evidence is needed on different community based models of distributing HIVST that are responsive to the needs of community members and compliment facility based testing. In this paper, we highlighted and demonstrated the positive community opinions of and experiences with and the social implications of a door-to-door distribution approach of HIVST to show that door-to-door distributed HIVST by lay counsellors is highly acceptable. This approach to distribution of HIVST can therefore be an additional option that that can supplement other models of distributing HIVST. However, we also caution that it should be accompanied by counselling and active linkage to care to buffer any resulting social harms and facilitate access to HIV treatment.

## Data Availability

Dataset for this study can be requested from corresponding authors and PIs.

## Abbreviations

ART: Antiretroviral therapy
CHiPs: Community HIV care providers
CRT: Community randomised trial
HIV: Human immunodeficiency virus
HIVST: HIV self-testing
FGD: Focus Group Discussion
HTS: HIV testing services
IDI: In-depth interview
PopART: Population effects of HIV treatment
UTT: Universal Test and Treat
IFU: Information for use
